# Supplementary material for: Master Regulator Analysis of the SARS-CoV-2/Human Interactome
Source: J Clin Med. 2020 Apr 1;9(4):982. doi: 10.3390/jcm9040982 (PMC7230814; doi:10.3390/jcm9040982)
Supplement: Supplementary file 1 [file jcm-09-00982-s001.zip › TableS3_pangolin.pdf]

human&pangolin	bat	human&pangolin	bat	human&pangolin	bat
ALA16	THR16	HIS228	ARG228	LEU568	LYS568
GLU23	ASP23	SER254	PHE254	ALA569	THR569
THR27	MET27	ALA296	GLU296	VAL573	ILE573
GLU35	LYS35	VAL298	LEU298	GLY575	ASP575
TYR41	HIS41	GLN325	GLU325	LYS577	ARG577
ASN49	ASP49	GLU329	ASN329	ARG582	GLY582
THR55	ASN55	ASP367	GLU367	ASN586	LYS586
ASN63	ASP63	ILE407	VAL407	LYS596	GLN596
GLN86	GLU86	ALA412	VAL412	LYS600	ARG600
ALA99	ILE99	ILE421	MET421	ASN601	LYS601
SER106	PRO106	THR445	ASN445	PHE603	TYR603
THR118	SER118	GLN472	GLU472	ALA614	SER614
THR122	ALA122	GLU483	LYS483	LYS631	ASN631
ASN134	LYS134	TYR521	PHE521	VAL658	GLU658
ASN137	LYS137	GLN522	GLU522	ARG671	TRP671
SER170	ALA170	GLN526	HIS526	PHE684	HIS684
ALA193	GLY193	GLN531	ARG531	LYS689	GLY689
ASN194	TYR194	LYS534	GLN534	VAL691	LEU691
GLY205	ARG205	GLU536	ASP536	GLY751	ALA751
GLY211	GLU211	GLU549	ASP549	PHE762	ILE762
TYR215	PRO215	GLN552	LYS552	LYS769	ARG769
ILE223	MET223	LEU560	VAL560	LYS771	THR771
GLU224	LYS224	PRO565	ALA565	ALA782	SER782
