# Supplementary figures and images for: Master Regulator Analysis of the SARS-CoV-2/Human Interactome
Source: J Clin Med. 2020 Apr 1;9(4):982. doi: 10.3390/jcm9040982 (PMC7230814; doi:10.3390/jcm9040982)

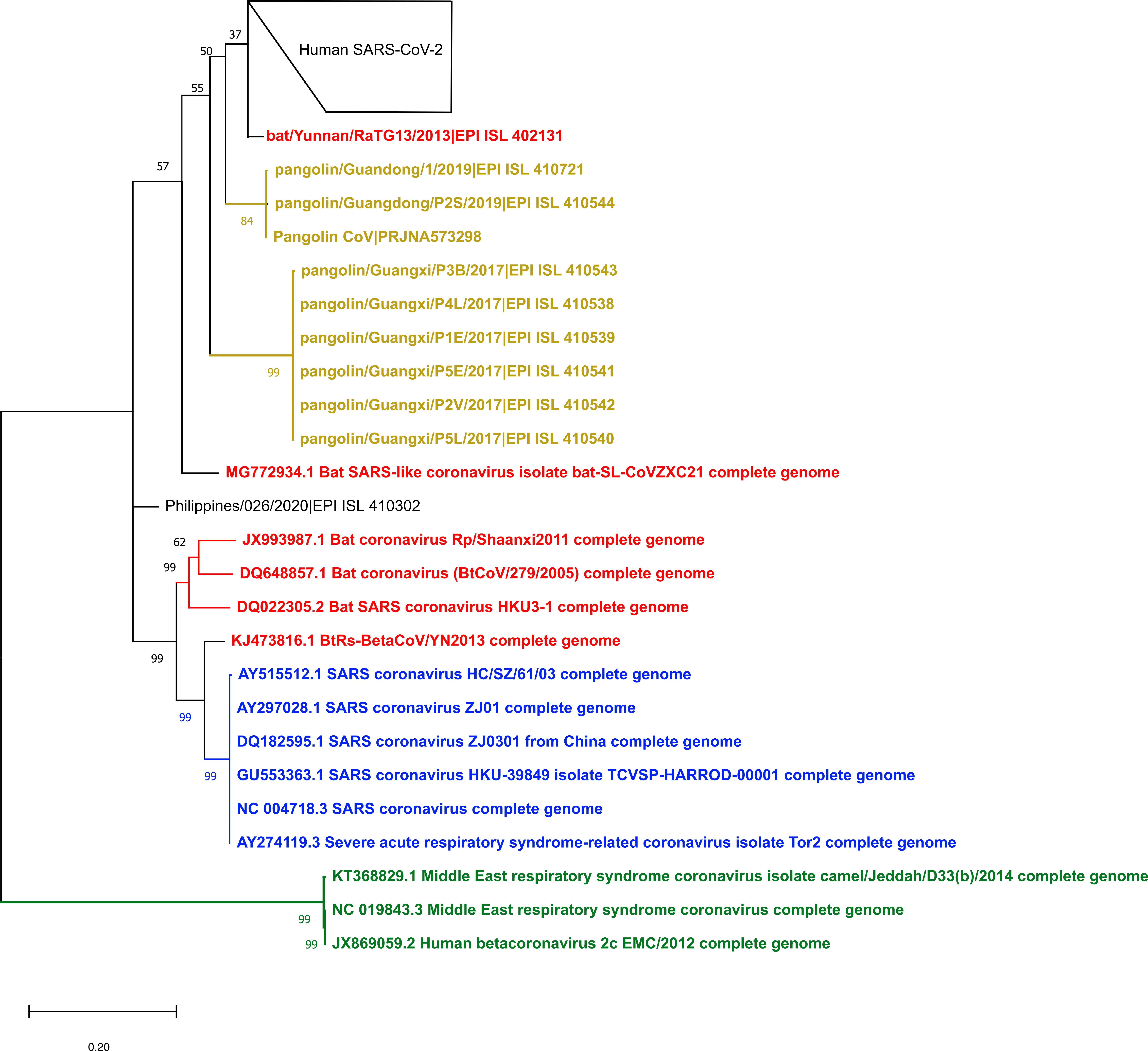

Supplement: Supplementary file 1 [file jcm-09-00982-s001.zip › FigureS1_tree.png]

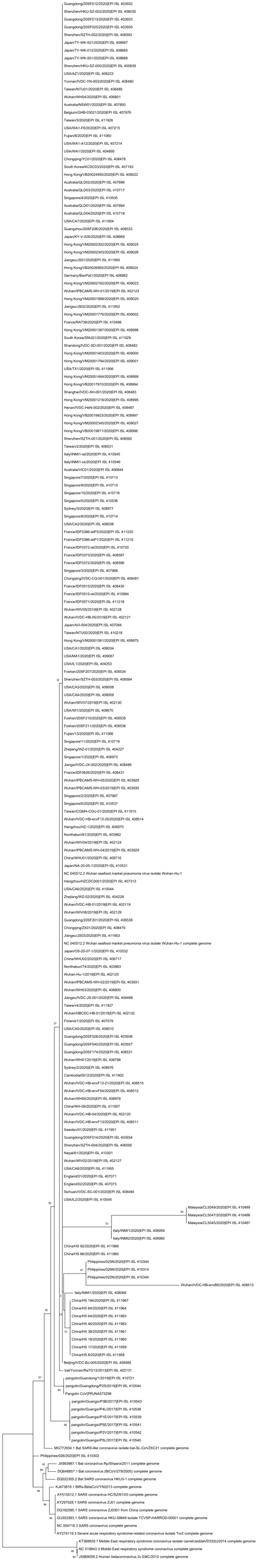

Supplement: Supplementary file 1 [file jcm-09-00982-s001.zip › FigureS2_fulltree.pdf]

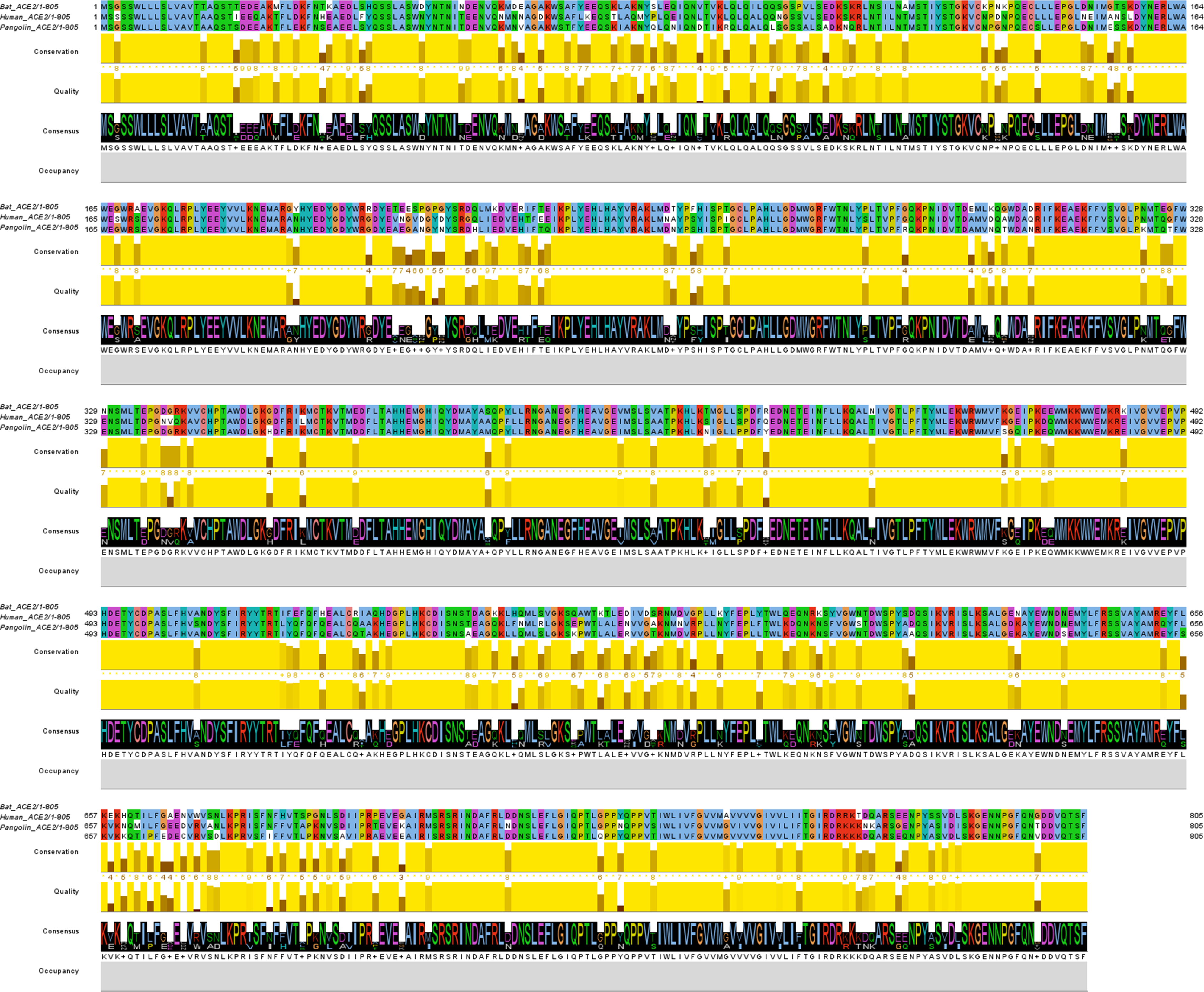

Supplement: Supplementary file 1 [file jcm-09-00982-s001.zip › FigureS3_msa.tif]
